# Symbiosis Contribution of Non-nodulating *Bradyrhizobium cosmicum* S23321 after Transferal of the Symbiotic Plasmid pDOA9

**DOI:** 10.1264/jsme2.ME22008

**Published:** 2022-06-08

**Authors:** Dyah Wulandari, Panlada Tittabutr, Pongpan Songwattana, Pongdet Piromyou, Kamonluck Teamtisong, Nantakorn Boonkerd, Pakpoom Boonchuen, Neung Teaumroong

**Affiliations:** 1 School of Biotechnology, Institute of Agricultural Technology, Suranaree University of Technology, Nakhon Ratchasima 30000, Thailand; 2 Molecular and Applied Microbiology Laboratory, Center of Research and Service, Diponegoro University 50275, Indonesia; 3 The Center for Scientific and Technological Equipment, Suranaree University of Technology, Nakhon Ratchasima 30000, Thailand

**Keywords:** nodulation, horizontal gene transfer, DOA9, transconjugant, chimeric

## Abstract

The symbiotic properties of rhizobial bacteria are driven by the horizontal gene transfer of symbiotic genes, which are located in symbiosis islands or on plasmids. The symbiotic megaplasmid pDOA9 of *Bradyrhizobium* sp. DOA9, carrying the *nod*, *nif*, *fix*, and type three secretion system (T3SS) genes, has been conjugatively transferred to different *Bradyrhizobium* strains. In the present study, non-nodulating *B. cosmicum* S23321, which shows a close phylogenetic relationship with *Bradyrhizobium* sp. DOA9, but lacks symbiotic properties, was used to carry pDOA9 (annotated as chimeric S2:pDOA9). The results obtained showed that pDOA9 conferred symbiotic properties on S23321; however, nodulation phenotypes varied among the DOA9, chimeric ORS278:pDOA9, and S2:pDOA9 strains even though they all carried symbiotic pDOA9 plasmid. S23321 appeared to gain symbiotic nodulation from pDOA9 by processing nodulation genes and broadening the host range. The present results also showed the successful formation of active nodules in *Arachis hypogaea* (Dalbergoid) and *Vigna radiata* (Millitoid) by chimeric S2:pDOA9, while *Crotalaria juncea* (Genistoid) and *Macroptilium atropurpureum* (Millitoid) formed nodule-like structures. The formation of nodules and nodule-like structures occurred in a nod factor-dependent manner because the nod factor-lacking strain (S2:pDOA9Ω*nodB*) completely abolished nodulation in all legumes tested. Moreover, T3SS carried by S2:pDOA9 exerted negative effects on symbiosis with *Crotalaria juncea*, which was consistent with the results obtained on DOA9. T3SS exhibited symbiotic compatibility with *V. radiata* when nodulated by S23321. These outcomes implied that pDOA9 underwent changes during legume evolution that broadened host specificity and the compatibility of nodulation in a manner that was dependent on the chromosomal background of the recipient as well as legume host restrictions.

Plasmids are important extrachromosomal, self-replicative DNA vehicles that may be vertically or horizontally transmitted. The genetic information carried on plasmids generally corresponds to accessory genes that have specific functions in processes, such as antibiotic resistance, detoxification, virulence, the catabolism of uncommon metabolites, and the capacity to invade tissues (Hall *et al.*, 2016). The symbiotic nodulation (*nod*) and nitrogen fixation (*nif/fix*) genes required for rhizobia to develop symbiosis with legumes and the symbiosis toolkit are generally found on a symbiotic plasmid or in a genomic island that may be transferred between strains by horizontal gene transfer (HGT) (Bedhomme *et al.*, 2017). Bradyrhizobia generally harbor symbiotic genes on genomic islands with a few exceptions, such as *Bradyrhizobium* sp. DOA9, which harbors a large symbiotic plasmid containing the *nod*, *nif*, and type three secretion system (T3SS) genes. This strain also contains *nif* genes on its chromosome (Okazaki *et al.*, 2015). The transconjugation of pDOA9 into photosynthetic *Bradyrhizobium* sp. ORS278 was successfully accomplished in a previous study. Symbiotic pDOA9 in chimeric ORS278:pDOA9 confounded the symbiotic relationship in the original host (*Aeschynomene indica* and *A. evenia*) and did not confer the type of symbiotic relationship found with DOA9 in a nod factor (NF)-dependent host (Songwattana *et al.*, 2019). These findings suggest that (i) the symbiosis tools required for classical nodulation render the symbiotic interaction incompatible in an NF-independent manner, and (ii) the symbiosis tools on pDOA9 do not confer all of the properties of the symbiotic relationship between ORS278 and legumes because some symbiotic factors may remain on the DOA9 chromosome (cDOA9), or incompatible factors may exist on the phylogenetically distant ORS278 chromosome. To prove that the symbiosis tools on pDOA9 are sufficient for the development of symbiosis and to investigate symbiotic contributions among *Bradyrhizobium* strains, non-nodulating *B. cosmicum* S23321, which is phylogenetically closely related to soybean symbionts, but lacks symbiosis islands (Okubo *et al.*, 2012; Okazaki *et al.*, 2015; Ormeno-Orrillo and Martinez-Romero, 2019), has been used as a new host harboring the DOA9 plasmid (pDOA9).

An examination of evolutionary relationships identified S23321 as the sister taxon of *B. cosmicum* 58S1, which is a symbiont of *Glycine max* cv. AC Orford. The internal nodes of *B. cosmicum* S23321 and 58S1 were located on the same branch as *B. lupini* USDA-3051 and *B. canariense* SEMIA-928, which are symbionts of *Lupinus agustifolius* and *Lupinus* sp., and, thus, shared the most recent common ancestor. However, an assessment of branch lengths showed that the *Lupinus* symbiont was older than S23321 (Wasai-Hara *et al.*, 2020). The close phylogenetic background of the S23321 chromosome with other bradyrhizobia strains implies that this strain has the potential for mutualism after receiving the symbiosis toolkit from neighboring strains.

To elucidate the functions of the symbiosis toolkit carried on pDOA9, the present study transferred pDOA9 into *B. cosmicum* S23321 using conjugative transfer. The nodulation properties of the new chimeric S23321 strain (S2:pDOA9) were investigated in all legumes that were originally nodulated by DOA9. We also examined symbiotic properties that depend on the *nod* and T3SS factors carried by pDOA9 plasmid. Therefore, pDOA9 lacking the *nodB* gene (pDOA9Ω*nodB*) and T3SS (pDOA9Ω*rhcN*) was also transferred into S23321 (S2:pDOA9Ω*nodB*, S2:pDOA9Ω*rhcN*), and nodulation was observed in legume tests.

## Materials and Methods

### Bacterial strains and culture media

The *Bradyrhizobium* strains used in the present study (Table 1) were cultured in arabinose-gluconate (AG) medium (Sadowsky *et al.*, 1987) at 28°C on a rotary shaker at 180‍ ‍rpm for 5 days. The *Escherichia coli* strain was cultured in Luria Bertani (LB) medium (Bertani, 1951) at 37°C on a rotary shaker at 180‍ ‍rpm for 18 h. The culture media of derivative mutants or recombinant plasmid-carrying strains were supplemented with appropriate antibiotics at the following concentrations: 20‍ ‍μg mL^–1^ cefotaxime (cefo), 50‍ ‍μg‍ ‍mL^–1^ nalidixic acid (nal), and 200‍ ‍μg mL^–1^ spectinomycin (spec).

### Construction of chimeric strains

Chimeric *B. cosmicum* S23321 carrying pDOA9 (S2:pDOA9) and its derived chimeric *nodB* mutant (S2:pDOA9Ω*nodB*) and *rhcN* mutant (S2:pDOA9Ω*rhcN*) strains were obtained by the triparental mating of *Bradyrhizobium* sp. ORS278:pDOA9, ORS278:pDOA9Ω*nodB*, or ORS278:pDOA9Ω*rhcN* (donor strain) with *B. cosmicum* S23321 (recipient strain) and *E. coli* HB101 carrying pRK2013 plasmid (helper strain) (Figurski and Helinski, 1979; Okazaki *et al.*, 2004) (Table 1). Briefly, 5-day cultures of chimeric *Bradyrhizobium* sp. ORS278, its derivatives, and *B. cosmicum* S23321 were washed with AG broth, and an 18-h culture of the *E. coli* HB101 (pRK2013) helper strain was washed with LB without the addition of antibiotics. Mixed cultures with a recipient:donor:helper ratio of 5:3:1 were dropped onto AG medium containing 10‍ ‍mM MgCl_2_ to facilitate better mating, followed by an incubation at 30°C for 3 days. Colonies grown on these plates were resuspended in AG and spread on AG supplemented with a mixture of the three antibiotics (spectinomycin, nalidixic acid, and cefotaxime). An incubation was performed under light to enhance the production of canthaxanthin (Lorquin *et al.*, 1993). This step allowed ORS278 with chimeric S23321 to be distinguished by an orange colony morphology (Fig. S1A). Colorless colonies of chimeric S23321 were repeatedly restreaked on AG plates containing spectinomycin, nalidixic acid, and cefotaxime. The colony morphology of the transconjugants was also observed and compared with those of S23321 and DOA9-WT (Fig. S1B).

Transconjugants were confirmed by colony PCR amplification using 2 pairs of specific primers for genes located on the S23321 chromosome (cS23321) (*nifA* and *bchL*) and 2 pairs of specific primers for genes located on the ORS278 chromosome (cORS278) (hypothetical protein and *lysE* transporter). pDOA9 plasmid was confirmed using specific primers for genes located on pDOA9, including *moeB*, *repA*, *trbG*, *nodA*, *nodB*, *nifD*, *hupK*, *nodD2*, *rhcN*, and GAJ3851 (Table 1). The DNA templates of all transconjugants were prepared by a colony suspension after the following three thermal cycling steps: 10‍ ‍min at 98°C, followed by 10 cycles of 98°C for 2‍ ‍min and 4°C for 30 s. One microliter of the cell suspension was used as the template for PCR amplification. The cycling conditions of the PCR program were as follows: 5‍ ‍min at 95°C for activation, followed by 35 cycles of 95°C for 30‍ ‍s for denaturation, 55°C for 30‍ ‍s for annealing, and 72°C for 30‍ ‍s for elongation, with a final elongation cycle of 72°C for 10‍ ‍min. PCR amplicons were examined by 1% TAE agarose gel electrophoresis run at 100‍ ‍V for 30‍ ‍min.

### Plasmid stability test

The plasmid stability test was performed as described by Songwattana *et al.* (2019). In this experiment, chimeric S2:pDOA9 and its derivatives (S2:pDOA9Ω*nodB* and S2:pDOA9Ω*rhcN*) were cultured for 1‍ ‍week in AG broth in the absence of antibiotic selection and then spread on AG agar. The colonies that appeared were transferred onto AG agar containing a mixture of the antibiotics (200‍ ‍μg mL^–1^ spectinomycin, 50‍ ‍μg‍ ‍mL^–1^ nalidixic acid, and 20‍ ‍μg mL^–1^ cefotaxime) to confirm pDOA9. We also investigated whether a specific gene marker of pDOA9 plasmid was amplified by PCR.

### Assessment of nitrogen fixation in chimeric versus wild-type DOA9 in free-living stages

The efficiency of nitrogenase activity in the free-living stage was measured in the DOA9, S23321, and chimeric S2:pDOA9 strains. They were grown under microaerobic conditions in 10-mL test tubes (BD Vacutainer) containing 2‍ ‍mL of buffered nodulation medium-broth (BNM-B) soft agar (0.8% [w/v]) medium with 10‍ ‍mM succinate as the carbon source, supplemented with a cocktail of vitamins, in the presence or absence of 1 M NH_4_NO_3_ as the nitrogen source to assess nitrogenase enzyme activity under free-living conditions. Briefly, 5-day-old cultures were harvested by centrifugation at 4,000‍ ‍rpm for 15‍ ‍min. Cells were washed three times with sterilized BNM-B. Cell density was then measured by optical density at 600‍ ‍nm (OD_600_) using a spectrophotometer and adjusted to OD_600_=1 (approximately 1×10^8^‍ ‍cells‍ ‍mL^–1^). Bacterial cells were concentrated 15-fold and used as the starter for a mixed culture. Two milliliters of a mixed culture containing 1% (v/v) bacterial cells in BNM-B soft agar medium was transferred to a 10-mL tube with a rubber cap with three replications. Nitrogenase activity was measured by removing 1‍ ‍mL of air from the tube and subsequently injecting 1‍ ‍mL of acetylene. Acetylene was injected to achieve a final concentration of 10% (v/v). Cultures were incubated at 28°C for 7 days without shaking and were manually mixed every 2 days. Gas chromatography was performed to measure the peak heights of ethylene (C_2_H_4_) and acetylene (C_2_H_2_) with 1-mL gas samples collected from the bottles using a PE-alumina packed column with a 150°C injector, 200°C oven, and 50°C flame ionization detector (FID). Nitrogenase activity units were calculated according to Wongdee *et al.* (2016).

### Plant cultivation and symbiosis ana­lysis

The symbiotic efficiency and nodulation ability of transconjugants were tested using legumes, including members of the Genistoid tribe: *Crotalaria juncea*, the Dalbergioid tribe: *Aeschynomene americana*, *A. afraspera*, *Arachis hypogaea*, and *Stylosanthes hamata*, and the Milletioid tribe: *Vigna radiata* cv. SUT4 and *Macroptilium atropurpureum*. Seeds of *C. juncea*, *A. hypogaea*, and *V. radiata* cv. SUT4 were surface sterilized in 95% (v/v) ethanol for 10‍ ‍s before the addition of 3% (v/v) sodium hypochlorite to completely immerse the seeds. The seeds of *A. americana*, *A. afraspera*, *S. hamata*, and *M. atropurpureum* were surface sterilized in 95% (v/v) ethanol for 10‍ ‍s before adding 98% H_2_SO_4_ for an appropriate amount of time (Teamtisong *et al.*, 2014) until the pericarp of the seeds was no longer observable. Seeds were then rinsed ten times with sterilized water. After soaking in water overnight, sterilized seeds were placed on plates containing sterilized 0.8% (w/v) water agar and kept in the dark for 1–2 days. Germinated seeds of *A. hypogaea*, *V. radiata*, and *C. juncea* were grown in Leonard’s jars filled with sterilizing vermiculite (Somasegaran and Hoben, 1994). *A. americana*, *A. afraspera*, *S. hamata*, and *M. atropurpureum* were grown in a hydroponic system in 50-mL tubes filled with BNM medium (Ehrhardt and Atkinson, 1992). All plants were grown under controlled environmental conditions at a temperature of 28±2°C under a 16-h light/8-h dark cycle with a light intensity of 300 μE m^2^ S^–1^ and 50% humidity. Each seedling was inoculated with 1‍ ‍mL of a cell suspension at a density of approximately 1×10^8^‍ ‍cells‍ ‍mL^–1^ (OD_600_=1) 5 days after planting. Nodulation and nitrogen fixation abilities were measured at 21 days post-inoculation (dpi). Five plants were analyzed for plant dry weight, the number of nodules, and nitrogenase activity using an acetylene reduction assay (ARA) (Bonaldi *et al.*, 2011). Briefly, all nodules were collected from each plant, placed in headspace bottles with 10% (v/v) acetylene, and incubated at 28°C for 1 h. Gas chromatography was conducted to measure the peak heights of ethylene and acetylene with 1-mL gas samples from bottles under the same conditions indicated above. Experiments were performed with five replicates.

### Cytological ana­lysis

A cytological ana­lysis of nodules was performed as described by Songwattana *et al.*, 2019. Fresh nodules were sectioned at a thickness of 40–50‍ ‍μm using a VT1000S vibratome (Leica Nanterre). The plant cell walls of nodules were stained with 0.01% (w/v) calcofluor (plant staining dye) for 20‍ ‍min, followed by an incubation in a mixture of 30‍ ‍μM propidium iodine (PI) and 5‍ ‍μM Syto9 (1:1) in 50‍ ‍mM Tris-HCl buffer, pH 7.0 for 25‍ ‍min. Green Syto9 staining was used to identify living cells and red PI staining for dead cells (Haag *et al.*, 2011). After staining, sections were mounted and observed under a confocal laser scanning microscope (Olympus Fluoview FV1000). Calcofluor was excited with a 405-nm laser line and detected with a 460- to 500-nm emission filter. Syto9 was excited at 488‍ ‍nm and detected at 490–522‍ ‍nm. PI was excited at 535‍ ‍nm and detected at 617–636‍ ‍nm. Images were remodeled using NIS elements software (Nikon) and adjusted for publication purposes.

### Statistical ana­lysis

Data from all experiments were analyzed using SPSS software (SPSS versions 17.0 Windows; SPSS) (Bryman and Cramer, 2011) to confirm significant differences via an ana­lysis of variance (ANOVA) with the F test (*P*≤0.05). Post hoc testing was performed using Tukey’s HSD test at *P*≤0.05.

## Results

### Conjugative transfer of pDOA9 into non-nodulating *B. cosmicum* S23321

The success of all chimeric strains was confirmed by PCR using specific primers of the genes located on the chromosomes of S23321 (cS23321) and ORS278 (cORS278), and other genes located on pDOA9 (Fig. S2 and Table S1). In chimeric S2:pDOA9 and its derivatives (S2:pDOA9Ω*nodB* and S2:pDOA9Ω*rhcN*), the genes located on cS23321 and all genes on pDOA9 were detected, whereas those on cORS278 were not (Fig. S2). In pDOA9Ω*nodB* and pDOA9Ω*rhcN*, the *nodB* and *rhcN* genes were not detected in the corresponding mutant strains (Fig. S2). Moreover, the colony morphology of all chimeric strains was similar to that of S23321-WT (Fig. S1B). The stability of pDOA9 plasmid in S23321 was analyzed and the results obtained showed that 100% of the colonies derived from the chimeric and mutant chimeric strains S2:pDOA9; S2:pDOA9Ω*nodB* and S2:pDOA9Ω*rhcN* grew under antibiotic selection. Collectively, these results suggested that pDOA9 plasmid was successfully transferred into non-nodulating S23321 by conjugative transfer and was stably maintained in the new *Bradyrhizobium* transconjugant strain.

### The transfer of pDOA9 increased the ability of the chimeric strain to fix N_2_ in the free-living stage

**Fig. 1.**
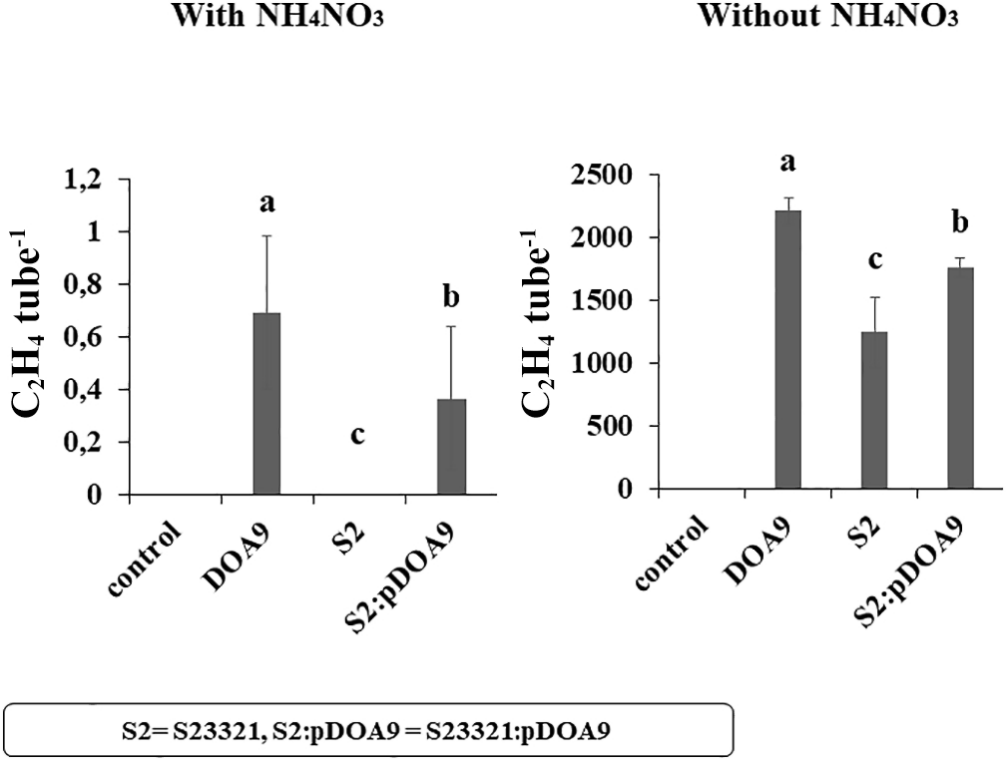
Nitrogenase activity measured via the acetylene reduction assay (ARA) in *Bradyrhizobium* sp. DOA9 (DOA9), S23321 (S2), and chimeric S23321 (S2:pDOA9) cultivated under free-living conditions with or without the nitrogen source (NH_4_NO_3_). The medium without bacterial cells was used as the control. Error bars represent the standard deviation (SD) (*n*=5). Different letters above the error bars indicate significant differences at *P*<0.05 (Tukey’s HSD test).

The growth of the free-living stages of S23321, DOA9, and the chimeric S2:pDOA9 strain under microaerobic conditions in the absence of the N source affected nitrogenase activity, which was higher in all strains than in the presence of the N source (NH_4_NO_3_) (Fig. 1). The presence of the N source reduced nitrogenase activity under all treatments; no activity was detected in S23321, while very low activity of less than 1‍ ‍nmol ethylene culture^–1^ was noted for DOA9-WT and S2:pDOA9. The results obtained in the absence of the N source indicated that DOA9-WT showed the highest nitrogenase activity of 2,205.2‍ ‍nmol ethylene culture^–1^ under free-living conditions, whereas that of S23321 was the lowest at 1,243.1‍ ‍nmol ethylene culture^–1^, and the activity of S2:pDOA9 was significantly higher (1,761.0‍ ‍nmol ethylene culture^–1^) than that of S23321. These results implied that the transfer of pDOA9 enhanced the ability of S23321 to fix N_2_ in the free-living stage.

### Restoration of S23321 properties by the acquisition of symbiosis tools

**Fig. 2.**
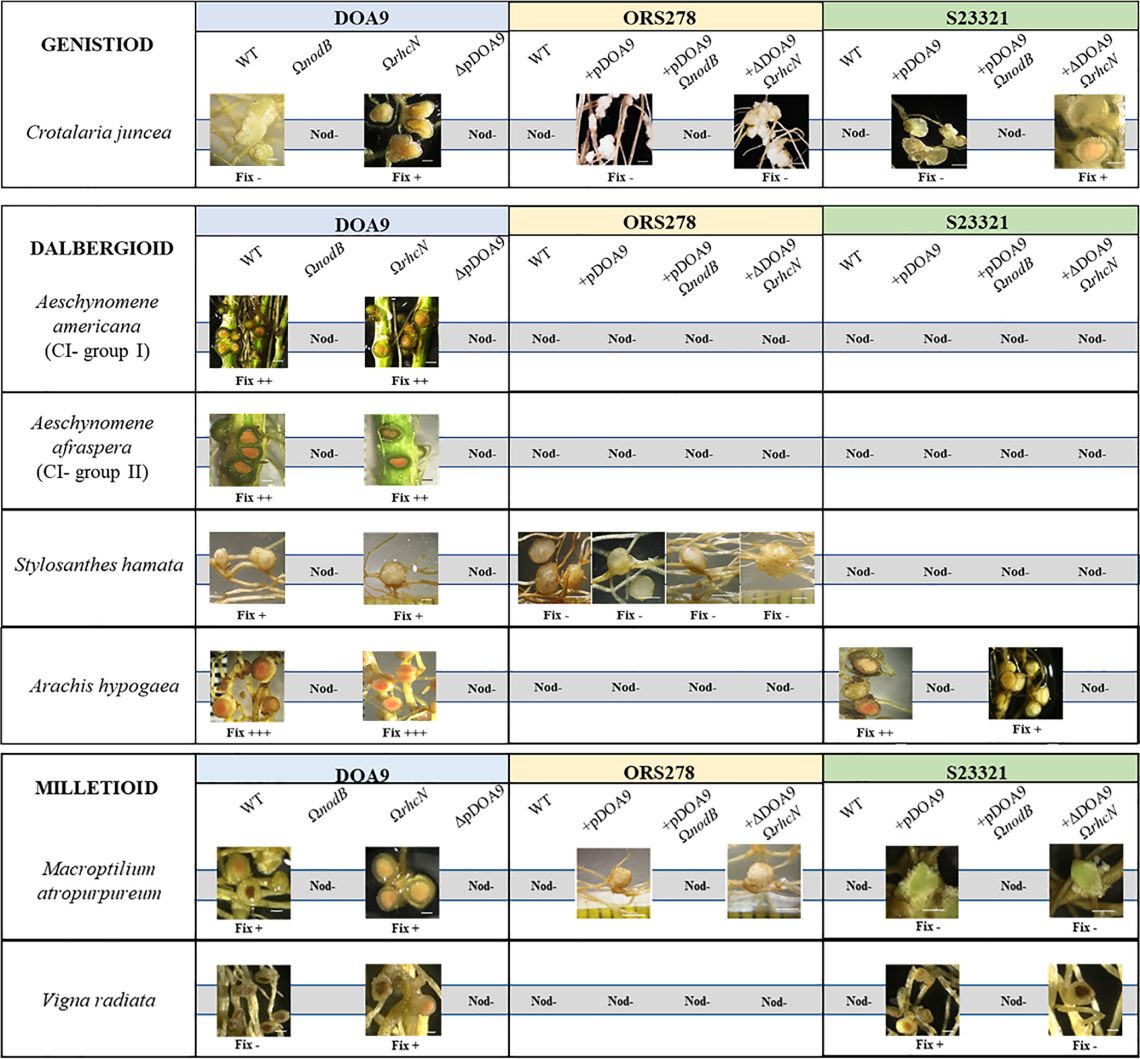
Summary of symbiotic properties of *Bradyrhizobium* sp. DOA9, ORS278, and *Bradyrhizobium cosmicum* S23321 wild-type (WT) strains and their derivative strains, including Ω*nodB*, Ω*rhcN*, ΔpDOA9 (curing plasmid), chimeric ORS278:pDOA9, ORS278:pDOA9Ω*nodB*, ORS278:pDOA9Ω*rhcN*, chimeric S23321:pDOA9, S23321:pDOA9Ω*nodB*, and S23321:pDOA9Ω*rhcN*. Legume tests of the members of four tribes, included the genistoid: *Crotalaria juncea*, dalbergioid: *Aeschynomene americana*, *Aeschynomene afraspera*, *Stylosanthes hamata*, and *Arachis hypogaea*, and milletioid: *Macroptilium atropurpureum* and *Vigna radiata*. The characteristics of symbiotic nodulation in all plant tests were observed at 21 dpi and indicated as follows: Nod–: no nodule formation; Fix–: inactive nodules with no nitrogenase activity; Fix+, Fix++, and Fix+++: active nodules with low, medium, and high nitrogenase activity, respectively.

**Fig. 3.**
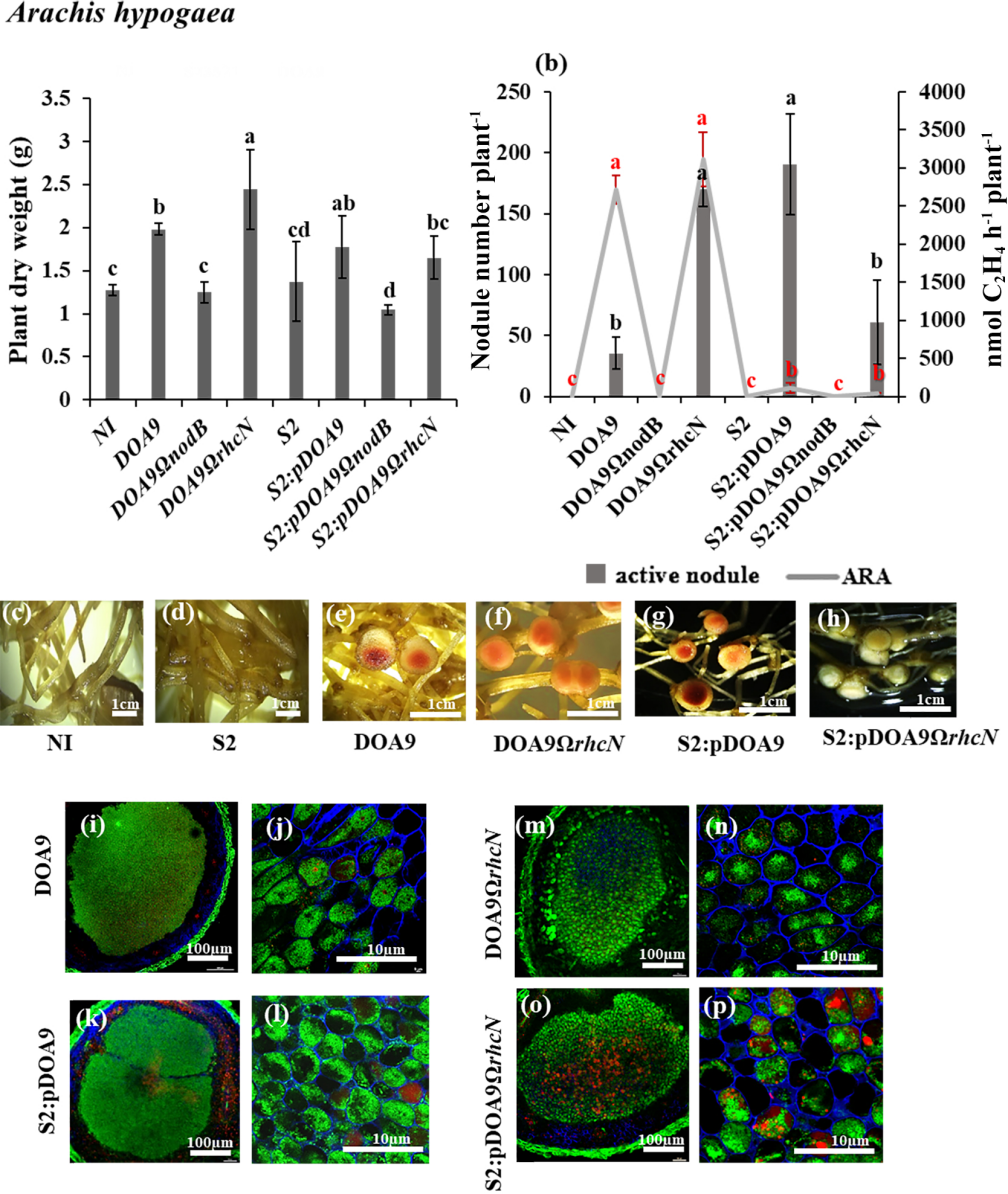
Symbiotic properties of *Bradyrhizobium cosmicum* S23321 (S2) and *Bradyrhizobium* sp. DOA9 strains and their derivatives in *Arachis hypogaea* (a, b, c, d, e, f, g, h, i, j, k, l, m, n, o, and p) after 21 dpi. Plant dry weight (a). The numbers of active (dark grey bar) and inactive nodules (light grey bar) per plant and nitrogenase activity (nmol C_2_H_4_ h^–1^ plant^–1^) are indicated by the grey line (b). Error bars in (a) and (b) represent the standard deviation (SD) (*n*=5). Different letters above the error bars indicate significant differences at *P*<0.05 (Tukey’s HSD test). In Fig. 3b, the error bars and letters above the error bars in black indicate the nodule number plant^–1^, while those in red indicate the ARA value. Nodule phenotypes (c, d, e, f, g, and h) and a cytological ana­lysis of nodules under confocal microscopy (i, j, k, l, m, n, o, and p). The scale bars in (c) to (h) indicate 1‍ ‍cm; those in i, k, m, and o indicate 100‍ ‍μm; and those in j, l, n, and p indicate 10‍ ‍μm. (Note that S2 refers to the S23321 strain)

**Fig. 4.**
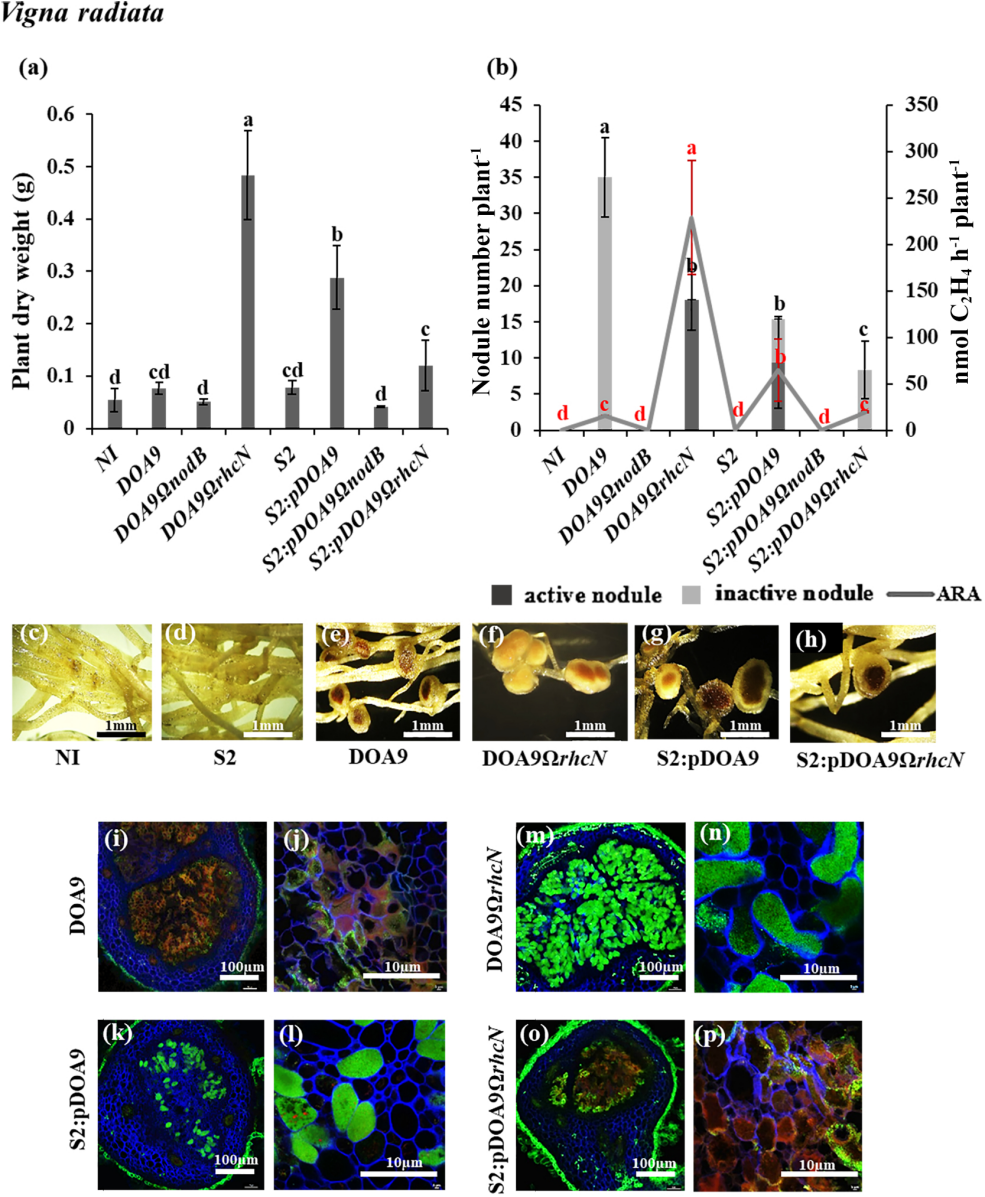
Symbiotic properties of *Bradyrhizobium cosmicum* S23321 (S2) and *Bradyrhizobium* sp. DOA9 strains and their derivatives in *Vigna radiata* (a, b, c, d, e, f, g, h, i, j, k, l, m, n, o, and p) after 21 dpi. Plant dry weight (a). The numbers of active (dark grey bar) and inactive nodules (light grey bar) per plant and nitrogenase activity (nmol C_2_H_4_ h^–1^ plant^–1^) are indicated by the grey line (b). Error bars in (a) and (b) represent the standard deviation (SD) (*n*=5). Different letters above the error bars indicate significant differences at *P*<0.05 (Tukey’s HSD test). In Fig. 4b, the error bars and letters above the error bars in black indicate for nodule number plant^–1^, and those in red indicate the ARA value. Nodule phenotypes (c, d, e, f, g, and h) and a cytological ana­lysis of nodules under confocal microscopy (i, j, k, l, m, n, o, and p). The scale bars in (c) to (h) indicate 1‍ ‍mm; those in i, k, m, and o indicate 100‍ ‍μm; and those in j, l, n, and p indicate 10‍ ‍μm. (Note that S2 refers to the S23321 strain)

To prove that the transfer of pDOA9 plasmid into S23321 conferred symbiotic properties in a classical NF-dependent manner, chimeric S2:pDOA9 was analyzed in 7 plants nodulated by DOA9. The first unexpected result was that S2:pDOA9 did not permit symbiotic interactions with *S. hamata* and two NF-dependent *Aeschynomene* species (*A. americana* and *A. afraspera*) (Fig. 2). S2:pDOA9 induced the formation of nodule-like structures in *C. juncea* and *M. atropurpureum*, which are phylogenetically distant (Fig. 2). However, S2:pDOA9 successfully nodulated *A. hypogaea* and *V. radiata* (Fig. 3 and 4).

The plant phenotype of *A. hypogaea* after 21 dpi showed that only DOA9-WT exhibited compatible symbiosis. Plant dry weight did not significantly differ between the DOA9-WT and S2:pDOA9 treatments (Fig. 3a), whereas the number of active nodules induced by chimeric S2:pDOA9 was higher (190 nodules plant^–1^) than that by DOA9-WT (35 nodules plant^–1^). However, active nodules derived from S2:pDOA9 were smaller than those from DOA9 (Fig. 3e and g) and also exhibited lower nitrogenase activity (Fig. 3b). These results implied that the nodules induced by S2:pDOA9 were small and less active because they were poorly developed and affected the ability to fix nitrogen, which did not reach the optimum level; 110.7‍ ‍nmol ethylene h^–1^ plant^–1^ under the S2:pDOA9 treatment, which was lower than that under the DOA9-WT treatment (2,717.3‍ ‍nmol ethylene h^–1^ plant^–1^) (Fig. 3b). Confocal observations showed that the bacteroid cells of S2:pDOA9 all resided in symbiosomes. DOA9-WT and S2:pDOA9 both resulted in the emission of green fluorescence by bacteroids, indicating the presence of living bacteroids inside the nodule (Fig. 3j and l). However, the plant infected by chimeric S2:pDOA9 showed the phenotype of nitrogen fixation by symbiotic nodules on 35 dpi (data not shown).

In *V. radiata* cv. SUT4 (Milletioid), inoculated plants displayed positive responses to the chimeric strains (Fig. 4). The plant dry weight of plants infected with S2:pDOA9 was significantly higher than that of plants infected with DOA9-WT (Fig. 4a). The assessment of nodulation efficiency showed that DOA9-WT induced the greatest number of defective nodules in *V. radiata* with low nitrogenase activity (Fig. 4b and e). The bacteroids inside DOA9 nodules stained red, indicating that the majority of cells were dead (Fig. 4i and j). Nevertheless, the S2:pDOA9 inoculation significantly reduced nodule numbers, with a mixture of active and inactive phenotypes (Fig. 4b and g). However, the bacteroids inside S2:pDOA9 nodules were alive and active (Fig. 4k and l). Therefore, the evaluation of nitrogen fixation ability revealed that plants infected by S2:pDOA9 showed significantly higher nitrogenase activity than those infected by DOA9-WT (Fig. 4b).

**Fig. 5.**
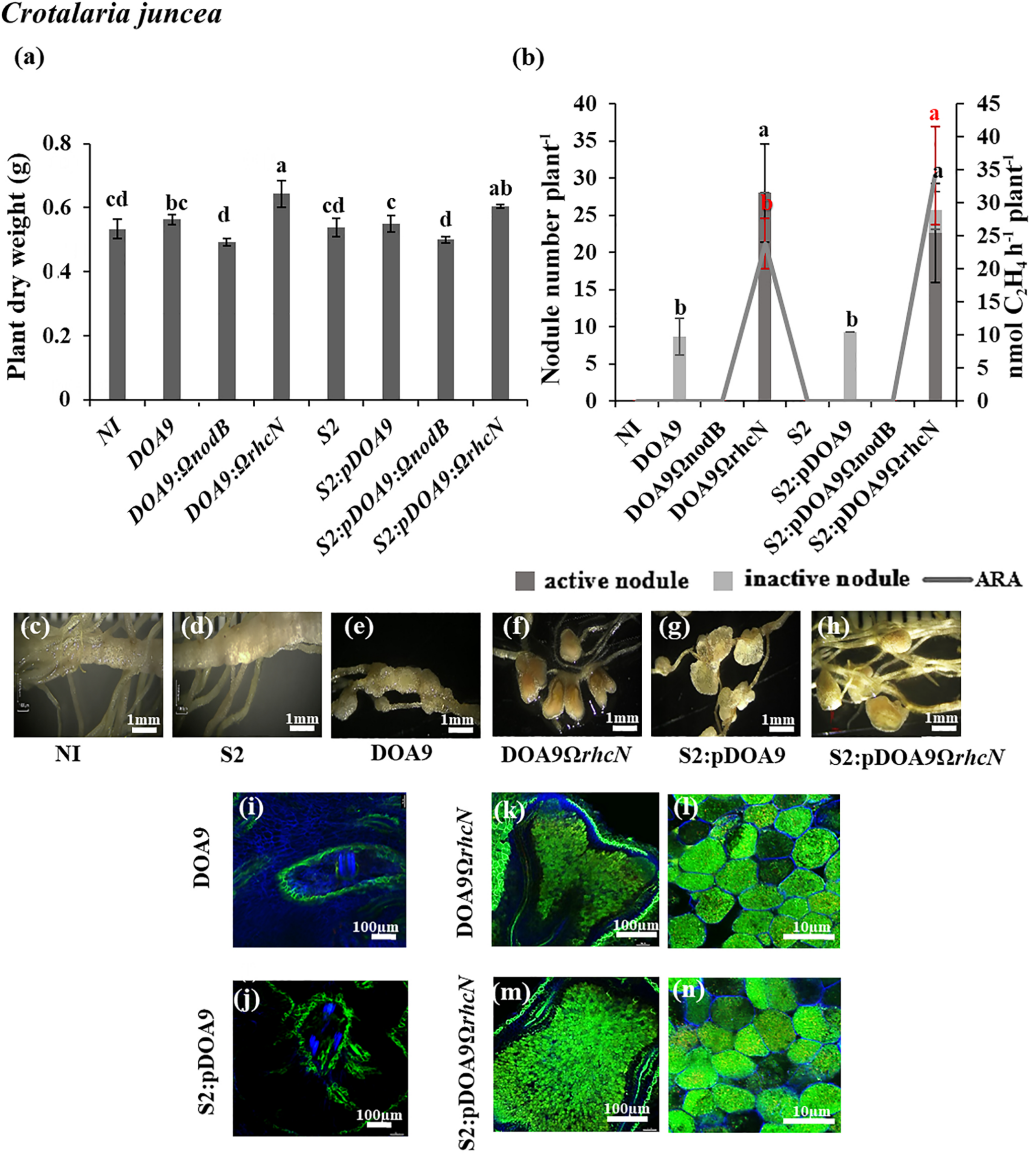
Symbiosis properties of *Bradyrhizobium cosmicum* S23321 (S2) and *Bradyrhizobium* sp. DOA9 strains and their derivatives in *Crotalaria juncea* (a, b, c, d, e, f, g, h, i, j, k, l, m, and n) after 21 dpi. Plant dry weight (a). The numbers of active (dark grey bar) and inactive nodules (light grey bar) per plant and nitrogenase activity (nmol C_2_H_4_ h^–1^ plant^–1^) are indicated by the grey line (b). Error bars in (a) and (b) represent the standard deviation (SD) (*n*=5). Different letters above the error bars indicate significant differences at *P*<0.05 (Tukey’s HSD test). In Fig. 5b, the error bars and letters above the error bars in black indicate for nodule number plant^–1^, and those in red indicate the ARA value. Nodule phenotypes (c, d, e, f, g, and h) and cytological analysis of the nodules under confocal microscopy (i, j, k, l, m, and n). The scale bars in (c) to (h) indicate 1‍ ‍mm; those in i, j, and k, and m indicate 100 µm; and those in l and n indicate 10 μm. (Note that S2 refers to the S23321 strain)

In *C. juncea*, the nodulation phenotype of S2:pDOA9 was similar to the plant inoculated by DOA9-WT. The plant dry weight of the DOA9-WT and S2:pDOA9 treatments did not significantly different from that of non-inoculated plants (Fig. 5a), and nitrogenase activity was not detected (Fig. 5b). The nodule phenotypes derived from DOA9-WT and S2:pDOA9 were both characterized as nodule-like structures (Fig. 5e and g) with the absence of the central infection area (Fig. 5i and j). This result indicated that the nodule organogenesis program was active in the early stage of infection, but also that bacterial cells were blocked from infecting the inside of the nodule.

However, the mutation of the nodulation gene in the chimeric strain (S2:pDOA9Ω*nodB*) completely abolished nodule formation in all plant tests (Fig. 2, 3, 4, and 5). This result demonstrated that nodulation by the S2:pDOA9 inoculation was induced in a NF-dependent manner.

### Effects of T3SS restrict the effectiveness of nodulation

Symbiotic compatibility between legumes and *Bradyrhizobium* species is often controlled by the function of T3SS. Previous studies showed that T3SS located on pDOA9 played a negative role in symbiosis with *C. juncea*, *A. hypogaea*, and *V. radiata* (Songwattana *et al.*, 2017). Therefore, we hypothesized that T3SS on pDOA9 may be responsible for impaired S2:pDOA9 symbiosis and be involved in the efficiency of the T3SS-dependent nodulation of legumes (*C. juncea*, *A. hypogaea*, and *V. radiata*). Moreover, T3SS may impair the symbiotic interaction with NF-dependent *Aeschynomene* species (*A. americana* and *A. afraspera*) and *S. hamata*, which were not nodulated by S2:pDOA9. Therefore, a T3SS-lacking plasmid was introduced into S23321 (S2:pDOA9Ω*rhcN*).

In *C. juncea*, S2:pDOA9Ω*rhcN* induced infected nodules and significantly increased plant dry weight (Fig. 5a), nodule numbers, and nitrogen fixation ability (Fig. 5b). The nodule phenotypes of S2:pDOA9Ω*rhcN*-infected plants were similar to those of the nodules resulting from the DOA9Ω*rhcN* inoculation. Centrally infected tissues and living bacteroids were detected (Fig. 5f, h, k, l, m, and n). These results implied that incompatible factors for *C. juncea* symbiosis were involved in T3SS located on pDOA9. S2:pDOA9Ω*rhcN* reduced the nodule number, plant dry weight, and nitrogen fixation in *A. hypogaea* (Fig. 3a and b) and *V. radiata* (Fig. 4a and b). Observations of the nodule phenotype under light and confocal microscopy showed brownish nodules and dead cells from the senescent region in the center of the nodules of *A. hypogaea* (Fig. 3h, o, and p) and *V. radiata* (Fig. 4h, o, and p) after the inoculation with S2:pDOA9Ω*rhcN*.

Moreover, chimeric S2:pDOA9Ω*rhcN* did not enhance the nodulation ability of chimeric S2:pDOA9 in NF-dependent *Aeschynomene* species (*A. americana* and *A. afraspera*) or *S. hamata*. This result suggested that the symbiotic relationship between S2:pDOA9 and these plants was not impaired by T3SS, but may require other factors that were absent from the S23321 chromosome.

## Discussion

### Enhancements in the nitrogen-fixing ability of S23321 by pDOA9

Bacterial adaptation or evolution is often accelerated by the acquisition of symbiosis tools located in external plasmids or symbiosis islands through horizonal gene transfer (HGT). In the present study, we investigated the conjugative transfer of symbiotic plasmids containing the *nod* and T3SS genes between *Bradyrhizobium* strains that are phylogenetically closely related. The recipient strain *B. cosmicum* S23321 is a non-nodulating *Bradyrhizobium* strain with a genome size of 7,231,841 bp (AP012279). This strain is phylogenetically closely related to *B. diazoefficiens* USDA110, but lacks symbiosis islands in its genome. However, the S23321 genome contains a complete *nif/fix* cluster including a single copy of the homocitrate synthase *nifV* gene in the operon and includes genes encoding a complete photosynthetic system. The *nif/fix* and photosynthetic genes are closely related to photosynthetic *Bradyrhizobium* (Okubo *et al.*, 2012). Moreover, the similarity and gene arrangement of the *nif/fix* cluster of S23321 is similar to that of the cluster on the DOA9 chromosome (cDOA9) (Fig. S3). In the free-living stage, S23321 fixed nitrogen in the absence of a nitrogen source (Fig. 1). This result is consistent with those obtained for other *nifV*-containing *Bradyrhizobium* strains, namely, the *nifV* gene of DOA9 was essential for nitrogen fixation under free-living conditions. The *nifV* gene of *Bradyrhizobium* sp. SUTN9-2 and ORS285 plays an important role in establishing nitrogen-fixing symbiosis with some plants (Hashimoto *et al.*, 2019). Moreover, the amino acid sequence encoded from the *nifV* gene of S23321 (BAL77793.1) shows 88.55% similarity to the NifV protein of DOA9 (BRADOA9_v1_51508). Therefore, S23321 has the potential to rapidly change its behavior from a free-living state to a legume endosymbiont. An examination of the contribution of symbiotic pDOA9 plasmid to S23321 revealed that the nitrogenase activity of S23321 increased in the absence of a nitrogen source (Fig. 1). This result indicated that pDOA9 harboring *nif/fix* genes enhanced the nitrogenase activity of S2:pDOA9 by increasing the copy number of *nif/fix* genes, particularly the regulatory *nifA* and *nifHDK* genes. Previous findings showed that the regulatory *nifA* gene of DOA9 was responsible for initiating the transcription of other genes involved in nitrogen fixation. *nifA* in the chromosome (*nifAc*) and *nifA* in the plasmid (*nifAp*) of DOA9 both function under symbiotic conditions, while *nifAc* and *nifAp* are indispensable for nitrogen fixation under free-living conditions and symbiotic conditions, respectively (Wongdee *et al.*, 2018). Based on its nucleotide sequence, the *nifA* gene of S23321 showed 80.42% similarity with the *nifAc* gene in DOA9, while a lower similarity of 69.49% was found with the *nifAp* gene of DOA9 (Fig. S3). Moreover, the amino acid at the N terminus of the NifA protein of S23321 was completely different from the NifAp protein of DOA9. The N-terminal domain is expected to have a regulatory function, and its modification may induce changes in gene regulation under different conditions (Wongdee *et al.*, 2018). Based on these findings, we hypothesized that nitrogen fixation by S2:pDOA9 in free-living conditions may be facilitated by the regulatory *nifA* gene of S23321 and the *nifAp* gene of pDOA9, which may individually regulate the functions of the *nif/fix* genes in the cluster on the S23321 chromosome and pDOA9. Therefore, an examination of a mutation in the *nifA* gene or a structural alteration in the *nifHDK* gene in S23321 and pDOA9 in future studies will provide a more detailed understanding of the regulatory crosstalk between the *nif* genes of cS23321 and pDOA9.

S2:pDOA9 showed lower nitrogenase activity than DOA9-WT under free-living conditions. A comparison of the genes of different strains revealed that S23321 lacked several genes involved in nitrogen metabolism, such as nitrilase, L-asparaginase, asparagine synthase, glutamate dehydrogenase, and *nosZ* (responsible for the final step of denitrification) (Okubo *et al.*, 2016), which may contribute to its lower N_2_ fixation efficiency relative to DOA9-WT. A previous study demonstrated that glutamate dehydrogenase in *Bacillus macerans* played an important role in ammonia assimilation during N_2_ fixation (Kanamori *et al.*, 1987). The *nosZ* gene of *B. diazoefficiens* USDA110 is essential for controlling the level of nitrous oxide (N_2_O) that accumulates under a low O_2_ content, which supports the potential of N_2_ fixation in both free-living cells and symbiotic nodules (Sameshima-Saito *et al.*, 2006; Ruiz *et al.*, 2019). In the present study, enhanced N_2_ fixation in S2:pDOA9 may be attributed to the substitution of glutamate dehydrogenase A (*gdhA*, BRADOA9_v1_p0329), which was present in pDOA9, but was absent in the S23321 genome (Fig. S3).

### Partial rescue of the symbiotic properties of chimeric S2:pDOA9

Nodulation properties varied among the DOA9, chimeric ORS278:pDOA9, and S2:pDOA9 strains, even though they all carried symbiotic pDOA9 plasmid. Unexpectedly, S2:pDOA9 was unable to nodulate 2 species of *Aeschynomene* (*A. americana* and *A. afraspera*) (Fig. 2). This result may be due to failed host plant recognition and a lack of some of the key factors required from cDOA9. These results are consistent with previous findings on chimeric ORS278:pDOA9 (Songwattana *et al.*, 2019). Although the main *nod* cluster responsible for NF biosynthesis is located on pDOA9, other genes related to nodulation are also present on the chromosome (cDOA9). For example, two-component respond regulators *nodVW*, DOA9 contains seven copies of the *nodV* gene (five on the chromosome and two on the plasmid) and 10 copies of the *nodW* gene (seven on the chromosome and three on the plasmid) (Okazaki *et al.*, 2015). Some copies of the *nodVW *gene on the DOA9 chromosome share high similarity with copies in *B. diazoefficiens* USDA110 (more than 85% identity), whereas the *nodV *(BDOA9_0203630) and *nodW* genes on the plasmid (BDOA9_0203420) share low similarity (lower than 67% identity). Two-component response regulators, NodVW protein are considered to recognize plant flavonoids that may positively regulate the transcription of other genes involved in nodulation or broaden the host range by increasing NF synthesis in combination with the NodD protein (Loh *et al.*, 2002). Moreover, multiple copies of *nolG* (6 copies) and *nodN* (2 copies) were found on cDOA9, but were absent on pDOA9. *nolG* and *nodN* on cDOA9 share more than 90% identity with copies in *B. diazoefficiens* USDA110. Previous studies reported that *B. diazoefficiens* USDA110 nodulated the roots of *A. americana* and *A. afraspera* (Chaintreuil *et al.*, 2013; Nicoud *et al.*, 2021). Therefore, the defective nodulation of chimeric S2:pDOA9 on *nod*-dependent *Aeschynomene* species may limited by the absence of symbiotic determinants on the cDOA9.

Chimeric S2:pDOA9 formed active nodules in *V. radiata* and *A. hypogaea*. S2:pDOA9 formed active nodules in *V. radiata* cv. SUT4, while DOA9-WT induced small nodules with the development of necrotic zones in nodules (Fig. 4). We hypothesized that the negative factor was retained on cDOA9 or that the background chromosome of S23321 exerted strong positive effects on nodulation in *V. radiata* cv. SUT4. Collectively, these results implied that the level of symbiotic compatibility may be dependent on the‍ ‍chromosome background of *Bradyrhizobium* carrying pDOA9. S2:pDOA9 permitted symbiotic compatibility, where­as DOA9-WT did not, even though it carried the same symbi­otic plasmid, pDOA9.

In the case of *A. hypogaea* nodulation, S2:pDOA9 induced a higher number of nodules than DOA9-WT. Moreover, the nodulation of *A. hypogaea* by S2:pDOA9 appeared to be delayed and showed lower nitrogen fixation at 21‍ ‍dpi than that by DOA9 (Fig. 3b, e, and g). Based on these results, the nodulation ability of S2:pDOA9 is controlled by nodulation genes on pDOA9, signaling molecules mediating symbiotic nodulation in a nod factor (NF)-dependent manner. These results were confirmed by a mutation in the *nodB* gene of pDOA9 abolishing nodulation (Fig. 2 and 3). Taken together, these results indicate that symbiotic nodulation was mediated by an NF-dependent pathway; however, the level of compatibility may also depend on the chromosome composition of the pDOA9-carrying host. In addition to NF, exopolysaccharide (EPS), lipopolysaccharide (LPS), and capsular polysaccharide (KPS) have been shown to play important roles in establishing effective symbiosis (Janczarek *et al.*, 2015). In the present study, the nodulation of *A. hypogaea* by S2:pDOA9 appeared to be delayed. These results are consistent with previous findings on *R. leguminosarum* bv. *phaseoli* in which the mutation of genes involved in LPS biosynthesis delayed infection and induced an irregular symbiosome structure (Brown *et al.*, 2011). The late nodulation of S2:pDOA9 in *A. hypogaea* may be attributed to incompatible factors involved in LPS or other components from the S23321 chromosome.

Okazaki *et al.* (2015) suggested that the 16S rRNA phylogenetic relationship of *Bradyrhizobium* sp. DOA9 shared a higher degree of similarity with the chromosome of *B. cosmicum* S23321 than that of *Bradyrhizobium* sp. ORS278. In the present study, the S23321 chromosome was more similar to cDOA9 than to the ORS278 chromosome. Genome comparisons in a Venn diagram showed that DOA9 and S23321 shared 2,310 unique genes families, whereas DOA9 and ORS278 only shared 142 unique genes (Fig. S4A). This result was confirmed by the average nucleotide identity (ANI) value, which was higher for DOA9:S23321 (87.03%) than for DOA9:ORS278 (79.43%) (Fig. S4B). This number may affect the symbiotic compatibility of pDOA9 with the chromosome host (rhizobial strains). Moreover, some genes located external to the symbiotic regions of different rhizobia were reported to be involved in symbiotic interactions (Doin de Moura *et al.*, 2020), including specific surface polysaccharides, metabolism genes, and transporters that are important for symbiosis in various rhizobia. Based on previous findings, we compared several genes involved in surface polysaccharides (*e.g.* *Ips*, *kps*, and *ndv*), metabolism genes (*e.g.* *gln*, *ilv*,* hem*, and *leu*), and transporters (*e.g.* *mdt*, dct, and *pst*) with the corresponding genes in the chromosomes of DOA9 and ORS278. The results obtained showed that all of the genes from the S23321 chromosome shared more similarities with the corresponding genes on cDOA9 than with those on the ORS278 chromosome (Table S2). Therefore, the transferal of symbiotic tools into different rhizobial strains may be achieved if recipient strains have a close phylogenetically background with the donor strains. However, successful symbiosis requires the two partners to be compatible with each other in every stage of the development of symbiosis (Wang *et al.*, 2018). Host-specific adaptation argues against the environmental need for bacteroids in which specific genes are transcribed and translated. This is one of the reasons why rhizobia did not nodulate all legumes, which also depends on host restrictions.

### Effects of type three secretion system (T3SS) of chimeric S2:pDOA9ΩrhcN on legume tests

Besides NF, T3SS in some *Bradyrhizobium* strains plays an important role in symbiotic compatibility with legume species. The T3SS apparatus in *Bradyrhizobium* strains shares homology with plant and animal pathogenic bacteria. This system is responsible for the translocation of effector proteins (T3Es) directly into the host cell, which exert positive or negative effects on nodulation depending on the host plant (Staehelin and Krishnan, 2015). A previous study reported that T3SS of *Bradyrhizobium* sp. ORS3257 exerted negative effects on *V. radiata* symbiosis, but was required for symbiotic compatibility with *V. mungo* (Songwattana *et al.*, 2021).

Regarding DOA9, T3SS has been shown to exert negative effects on *V. radiata* and *C. juncea* by inducing necrotic nodules and nodule-like structures, respectively (Songwattana *et al.*, 2017). A previous study revealed that T3SS on pDOA9 blocked the establishment of NF-independent symbiosis (*A. indica* and *A. evenia*), but did not impact the NF-dependent symbiosis due to incompatibility (Songwattana *et al.*, 2019). In the present study, T3SS in pDOA9 was involved in NF-dependent symbiosis by S23321. The nodulation of *C. juncea* was enhanced by S2:pDOA9Ω*rhcN*, which was consistent with the results obtained on DOA9Ω*rhcN* (Fig. 5). In contrast to DOA9, the mutation of T3SS reduced the symbiotic efficiency of‍ ‍S23321 when nodulated in *A. hypogea* and *V. radiata* (Fig. 3 and 4). Previous findings showed that the effector secreted by T3SS induced plant defense responses by triggering plant immunity through direct or indirect interactions with resistance (R) proteins in the host plant‍ ‍(Okazaki *et‍ ‍al.*,‍ ‍2013, 2016). However, a genome ana­lysis of ORS278 and S23321 did not identify homolog genes involved in T3SS on their genomes. Collectively, these results suggest that the symbiotic compatibility or incompatibility phenotypes displayed by chimeric ORS278:pDOA9 and S2:pDOA9 may be derived from effector proteins encoded by T3SS on pDOA9. DOA9 chromosome contains putative effector proteins that may be regulated and secreted through T3SS on pDOA9 and exert contrasting effects on nodulation. Therefore, further studies are needed to identify the positive or negative factors secreted from T3SS on pDOA9 to obtain a more detailed understanding of the mechanisms constraining the interaction or shaping it into mutualistic symbiosis.

### Plasmid-chromosome crosstalk in chimeric S23321

The same plasmid will give rise to different traits associated with the specificity and compatibility of chimeric-legume symbiosis in the presence of different background chromosomes and among different host plants. S2:pDOA9 exerted a wide range of effects in each legume test (Fig. 2). Responses to the acquisition of the symbiotic plasmid in the chimeric S2:pDOA9 transconjugant were divided into three scenarios. The first type of response was that strictly selected in both the bacterial chromosomal and symbiotic gene backgrounds, such as those observed in *A. americana* and *A. afraspera* legume plants. The second type was strictly selected only in the symbiotic gene background, as observed in *M. atropurpureum*. The third type was extensively acquired in a T3SS-dependent manner, as observed in *V. radiata*, *C. juncea*, and *A. hypogaea*.

Recurrent adjustments to chimeric genes and their mutant forms are observed during plasmid transfer. Experimental and metabolic modeling ana­lyses indicated that alterations to the nodule environment depended on accessory plasmid genes (Ramachandran *et al.*, 2011; Barreto *et al.*, 2012; diCenzo *et al.*, 2016; Palmer *et al.*, 2016; Zahran, 2017; Yang *et al.*, 2020). pDOA9 possesses a unique characteristic from other symbiotic plasmids in 24 rhizobia, including four genera and 11 species. pDOA9 was designated as an outgroup located far from other plasmids based on a common gene ana­lysis of the symbiotic plasmid *nod* (*nodCIJ*) and *fix* (*fixABC*) genes (Wang *et al.*, 2018).

This discovery is fascinating and will drive future research that may provide insights to establish (i) whether the background chromosome of S23321 is more compatible with specific legumes and possesses negative factors/effectors relative to the chromosome of DOA9 and (ii) if the negative factors/effectors found on pDOA9 or the S23321 chromosome help to cope with these negative factors/effectors. These questions will be of interest in the further study of factors/effectors with positive and negative effects on nodulation in the original strain DOA9 and also factors that have undergone functional changes to broaden host specificity during the history of legume evolution. This information may then be transferred to the study of S23321 or other *Bradyrhizobium* strains carrying pDOA9plasmid.

## Conclusion

The present study is the first to report that non-nodulating *B. cosmicum* S23321 was transformed to induce endosymbiosis nitrogen-fixing nodules by the transferal of symbiotic plasmid pDOA9. Phylogenetic proximity between the DOA9 chromosome and the S23321 chromosome contributed to the achievement of nodulation by and the nitrogen fixation ability of chimeric S2:pDOA9. However, mutualistic symbiosis in all legume tests was not completely permitted by chimeric strains due to the unknown mechanism constraining the interaction, even though they all carried the same symbiotic tools.

## Citation

Wulandari, D., Tittabutr, P., Songwattana, P., Piromyou, P., Teamtisong, K., Boonkerd, N., et al. (2022) Symbiosis Contribution of Non-nodulating *Bradyrhizobium cosmicum* S23321 after Transferal of the Symbiotic Plasmid pDOA9. *Microbes Environ ***37**: ME22008.

https://doi.org/10.1264/jsme2.ME22008

## Supplementary Material

Supplementary Material

## Figures and Tables

**Table 1.** Bacterial strains and plant species used in the present study

**Strain**	**Characteristics**	**Reference**
*Bradyrhizobium* sp. DOA9	Non-photosynthetic strain, isolated from a paddy field using *A. americana* as the trap legume, a Nod-dependent strain	Noisangiam *et al.*, 2012
DOA9Ω*nodB*	*nodB* mutant of the DOA9 strain obtained by the integration of pVO155-npt2-cefo^r^- npt2-gfp; cefo^r^ km^r^	Songwattana *et al.*, 2019
DOA9Ω*rhcN*	*rhcN* mutant of the DOA9 strain obtained by the integration of pVO155-npt2-cefo^r^- npt2-gfp; cefo^r^ km^r^	Songwattana *et al.*, 2019
ORS278:pDOA9	Chimeric *Bradyrhizobium* sp. ORS278 (sp^r^) containing the pDOA9 plasmid (pDOA9-pK18mob-sacB-cefo^r^), sp^r^ km^r^ cefo^r^	Songwattana *et al.*, 2019
ORS278:pDOA9Ω*nodB*	Chimeric *Bradyrhizobium* sp. ORS278 (sp^r^) containing the pDOA9Ω*nodB* plasmid, sp^r^ km^r^ cefo^r^	Songwattana *et al.*, 2019
ORS278:pDOA9Ω*rhcN*	Chimeric *Bradyrhizobium* sp. ORS278 (sp^r^) containing the pDOA9Ω*rhcN* plasmid, sp^r^ km^r^ cefo^r^	Songwattana *et al.*, 2019
*Bradyrhizobium cosmicum* S23321	Non-photosynthetic strain, isolated from a paddy soil non-nodulating strain	Okubo *et al.*, 2012
S2:pDOA9	Chimeric *B. cosmicum* S23321 (sp^r^) containing the pDOA9 plasmid (pDOA9-pK18mob-sacB-cefo^r^), sp^r^ km^r^ cefo^r^	This study
S2:pDOA9Ω*nodB*	Chimeric *B. cosmicum* S23321 (sp^r^) containing the pDOA9Ω*nodB* plasmid, sp^r^ km^r^ cefo^r^	This study
S2:pDOA9Ω*rhcN*	Chimeric *B. cosmicum* S23321 (sp^r^) containing the pDOA9Ω*rhcN* plasmid, sp^r^ km^r^ cefo^r^	This study
*Escherichia coli* HB101 (PRK2013)	*E. coli* HB101 carrying the pRK2013 helper plasmid; mob+, tra+, km^r^	Figurski and Helinski, 1979

**Tribes**	**Plant**	**Source of reference**
Dalbergoid	*Aeschynomene americana*	Thailand
	*Aeschynomene afraspera*	LSTM, IRD, France
	*Arachis hypogaea (peanut)*	Thailand
	*Stylosanthes hamata*	Thailand
Millitoid	*Macroptilium atropurpureum*	Thailand
	*Vigna radiata* cv SUT4	Thailand
Genistoid	*Crotalaria juncea*	Thailand
