# Supplementary material for: Symbiosis Contribution of Non-nodulating Bradyrhizobium cosmicum S23321 after Transferal of the Symbiotic Plasmid pDOA9
Source: Microbes Environ. 2022 Jun 8;37(2):ME22008. doi: 10.1264/jsme2.ME22008 (PMC9530727; doi:10.1264/jsme2.ME22008)

(A) (B)

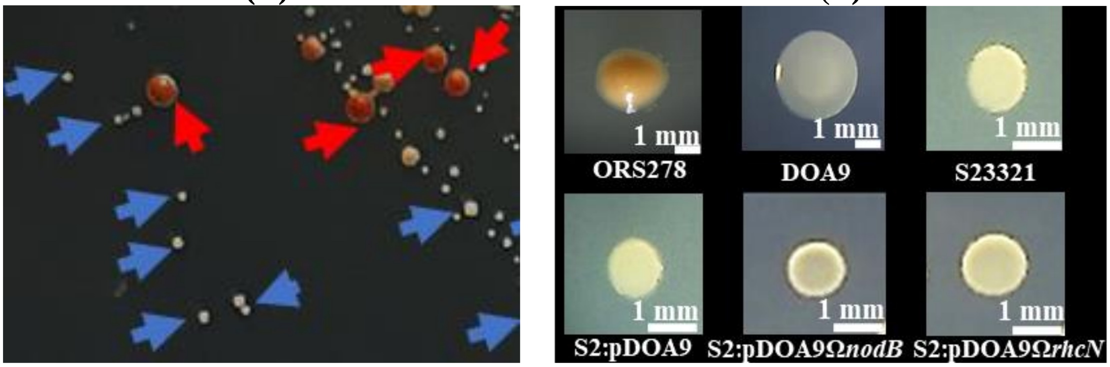

Fig. S1. Transconjugants were screened based on colony morphology using AG medium with antibiotics (spectinomycin 200 µg/ml, cefotaxime 20 µg/ml, and nalidixic acid 50 µg/ml). Red arrow = ORS278:pDOA9, blue arrow = chimeric S23321 (S2:pDOA9) (A). Colony morphology (B) of *Bradyrhizobium* sp. ORS278, *Bradyrhizobium* sp. DOA9, *B. cosmicum* S23321, S2:pDOA9, S2:pDOA9Ω*nodB*, and S2:pDOA9Ω*rhcN*. Wild-type strains were grown in AG medium, while chimeric strains and their derivatives were grown on AG medium with antibiotics.

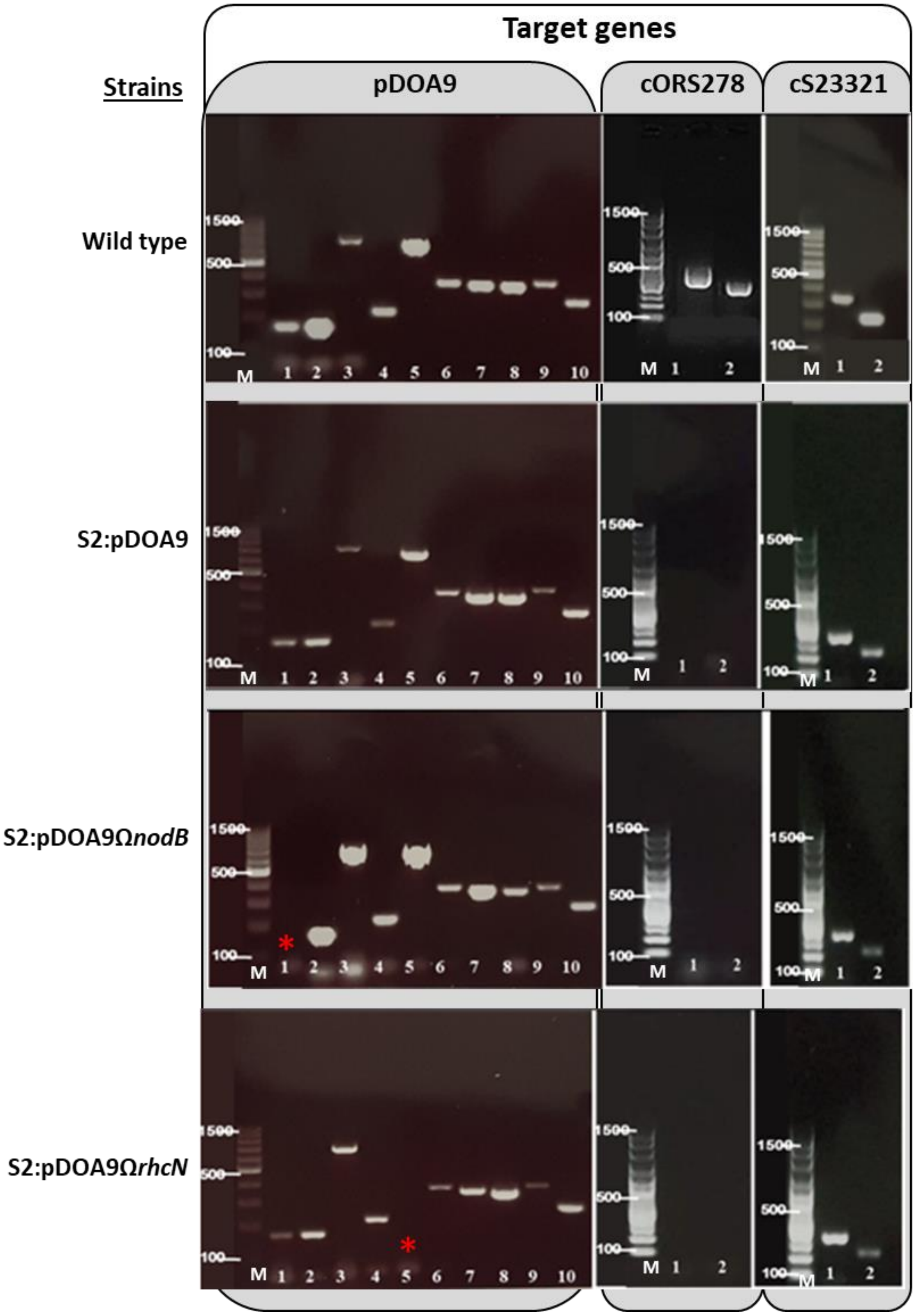

Fig. S2. The PCR products of chimeric strains S2:pDOA9, S2:pDOA9Ω*nodB*, and S2:pDOA9Ω*rhcN* were compared with those of the *B. cosmicum* S23321 and *Bradyrhizobium* sp. ORS278 wild type strains. PCR verification was performed using 10 specific primers for the genes located on the DOA9 plasmid (pDOA9), including *nodB* (Lane 1), *nodA2* (Lane 2), *nifDp* (Lane 3), *nodD2* (Lane 4), *rhcN* (Lane 5), *repA* (Lane 6), *moeB* (Lane 7), *trbG* (Lane 8), *HupK* (Lane 9) and GAJ38051 (Lane 10). The PCR products of genes located on the ORS278 chromosome (cORS278) were confirmed by using 2 specific primers for hypothetical proteins (Lane 1) and *lysE* transporters (Lane 2). The target genes located on the S23321 chromosome (cS23321) were detected by specific primers for *bchl* (Lane 1) and *nifA* (Lane 2). * Indicates the absence of the PCR products of the *nodB* and *rhcN* genes, which corresponded to the genotypes of the derivative chimeric S2:pDOA9Ω*nodB* and S2:pDOA9Ω*rhcN* mutant strains. The PCR products were observed on 1% TAE agarose gels. M=100 bp ladder (NEB, USA)

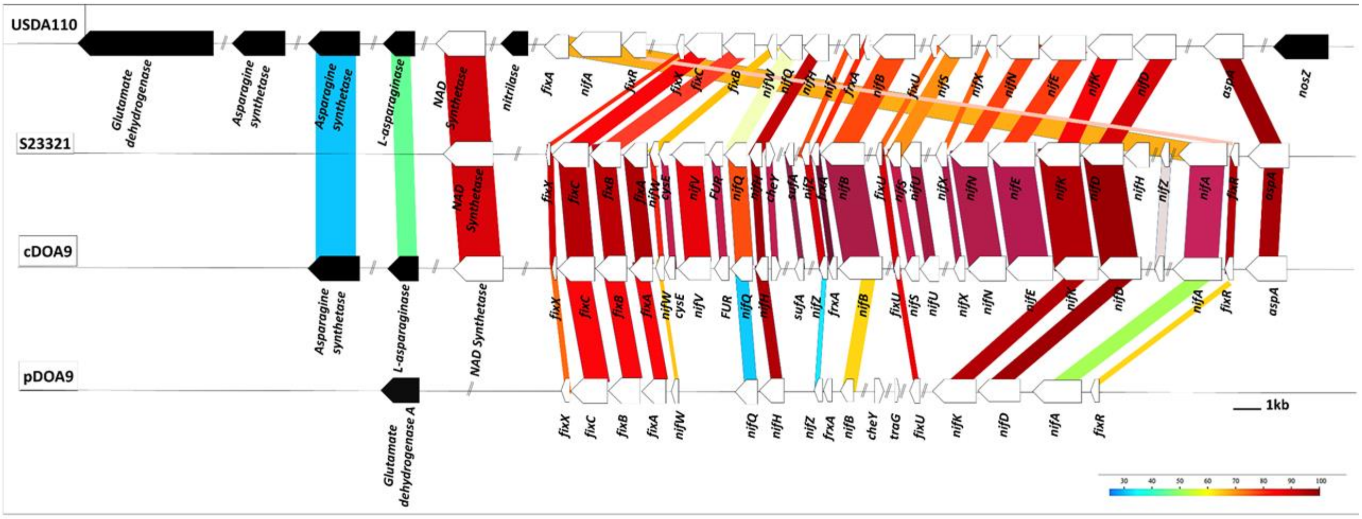

Fig. S3. Assessment of *nif/fix* gene organization in S23321 compared with the pDOA9 plasmid (pDOA9) and the chromosomes of DOA9 (cDOA9), and *B. diazoefficiens* USDA110 using GenomeMatcher. Black bars indicate genes absent in S23321. Double slash marks represent DNA regions that were not shown. Coloured strips represent the conserved gene regions among the compared strains, and the colour indicates the percent similarity.

A

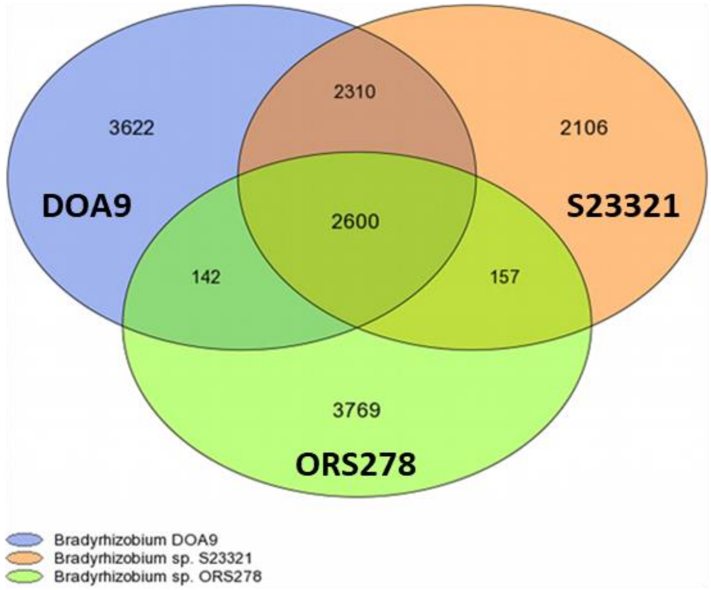

B

Metric	DOA9:S23321	DOA9:ORS278
Ortho ANI value (%)	87.03	79.43
DOA9 genome length (bp)	7,100,220	7,100,220
Query genome length (bp)	7,231,800	7,456,200

Fig. S4. Genome analysis of *Bradyrhizobium* sp. DOA9, S23321, and ORS278. A: Venn diagram representing the subgroups of a pan-genome of *Bradyrhizobium* sp. DOA9, S23321, and ORS278. Each set represents the gene families detected in a genome. The intersection of these sets represents the core genome which is 2600 families with 7957 genes. The number of gene families in the core genome corresponds to the size of the intersection. The fraction of genes corresponding to the accessory genome in DOA9 with S23321 is higher than in DOA9 with ORS278. It used the MicroScope gene families (MICFAM) parameter with 80% amino acid identity and 80% alignment coverage which are computed with the SiLiX software (Miele *et al*., 2011). B: The percentage of Average Nucleotide Identity (ANI) value indicates the comparison of DOA9:S23321 and DOA9:ORS278. The genome length (bp) of DOA9 (7,100,220) was compared with the query strains, including 7,231,800 bp of S23321 and 7,456,200 bp of ORS278.

Miele, V., Penel, S., and Duret, L. (2011) Ultra-fast sequence clustering from similarity networks from similarity networks with SiLiX. *BMC Bioinf* **12**: 116.

Table S1. The list of primers used for chimeric verification that specific to the S23321 chromosome, pDOA9 plasmid, and ORS278 chromosome and primers used for real-time PCR (qRT–PCR) specific to the S23321 chromosome.

Primer name	Sequence (5'-3')	References
**Primers**		
Primers for confirmation the S23321 chromosome		
bchL		
bchL-S23321.f	5'-CAAGCATGACTCGACCTTCA-3'	
bchL-S23321.r	5'-GCGAAGATCGAGTCGAAATCA-3'	
nifA		This study
nifA-S23321.f	5'-CATTGAGCGCACCGGTAAAC-3'	
nifA-S23321.r	5'-CATTGCGAGGATTGCTGGAGA-3'	
Primers for confirmation the pDOA9		
moeB		
moeB-252.f	5'- GAGCCGTCCTTCAGCAATTA-3'	
moeB-253.r	5'-GCGACTTCTCAGACCATCATAC-3'	
repA		
repA-250.f	5'-AAGAGAACGGCACCTTGTATG-3'	
repA-251.r	5'-TCCGAGGTCATCAGGAGAAA-3'	
trbG		
trbG-254.f	5'-CGTGTGTCACTTGAGACCAA-3'	
trbG-255.r	5'-GAAGGCGCGATAATTGAGGA-3'	
nodA2		
nodA2-196.f	5'-GGCGTTCAATGCAAGACC-3'	
nodA2-197.r	5'-GCGTACAATCCGAGTTCAG-3'	
nodB		
nodB-202.f	5'-GGTATGCGACAGAACATCCAC-3'	
nodB-203.r	5'-CGCACGTATATGTCTCAGCC-3'	
nifDp		Songwattana *et al.*, 2019
nifDp-4218.f	5'-GAGCCGAACGACCAACCA-3'	
nifDp-4219.r	5'-GCGTCGCCGCCGATATTGTAG-3'	
hupK		
hupK-256.f	5'- CGCAACGAGATCGACATCA-3'	
hupK-257.r	5'-GGTAGTCGCCTGCATCATT-3'	
nodD2		
nodD2-130.f	5'-GTCTTGCGAGCCCTTTACAC-3'	
nodD2-131.r	5'-CGCAAGCTGACATTTGAGAA-3'	
rhcN		
rhcN-4083.f	5'-GCAACAATCAGGCCATAATCAAG-3'	
rhcN-4084.r	5'-AGGGCATCTCCAATATGACGTTC-3'	
GAJ3851		
GAJ3851.f	5'-GCTCATGGGTGGGTTGAATA-3'	
GAJ3851.r	5'-TCCTGATTGGAACGGTTAAGG-3'	
Primers for confirmation the ORS278 chromosome		
Hypothetical protein		
Hp.f	5'-GATTGCCCATGGAATGGCAG-3'	
Hp.r	5'-CATAGCCTCTCTCTGGCAG-3'	
LysE		This study
LysE.f	5'-CAAAGGGAAACCCGATTGG-3'	
LysE.r	5'-CAATAGCGCGCTGAAGAAC-3'	
Primers for qRT-PCR		
**16S rRNA**		
16S.f	5'-ACTCCTACGGGAGGCAGCAG-3'	
16S.r	5'- ATTACCGCGGCTGCTGG-3'	
**O-antigen**		
O-antigen.f	5'-GTCAGCCCTTCAGGAAATTCGG-3'	
O-antigen.r	5'-CATGATTTCCGCGGGCTTC-3'	
**Glycosyltransferase**		
Gly.f	5'-GTTCCTGTTCGTCGTGTTGATC-3'	This study
Gly.r	5'-CATAACCATGGGTGTTGCGATAG-3'	
mdtA		
mdtA.f	5'-CATTCAGACCTTCGGAGACCAC-3'	
mdtA.r	5'-GTCTGTTCGCCTATGTGATCGG-3'	
mdtB		
mdtB.f	5'-GATGCCGATCAGCAAGACGAG-3'	
mdtB.r	5'-CTATGAGAGCTACATTCATCCGC-3'	

Table S2. Percentage similarity of genes involved in surface polysaccharide, metabolism, and transporter by comparison with the corresponding genes in chromosome of DOA9 (cDOA9) and ORS278 (cORS278) using standard nucleotide BLAST. The heat map shading in the table indicates the sequence similarity level of 0 to 100 percentage.

Percentage similarity by standard nucleotide BLAST			
	**Specific surface polysaccharide genes of cS23321**		
	**Description**	**cDOA9 (%)**	**cORS278 (%)**
lpsC	*Lipopolysaccharides*	93.33	76.9
Kps	*Capsular polysaccharide*	95.11	80.56
ndvB		92.9	78.97
ndvC	*Exopolysaccharide*	89.82	73.41
ndvA		94.35	83.89
	**Metabolism genes of cS23321**		
	**Description**	**cDOA9 (%)**	**cORS278 (%)**
glnB	Glutamine synthetase	100	99.11
glnK		100	96.3
ilvC	Isoleucine and valine synthesis	95.87	93.8
ilvD		97.39	91.81
hemA	5-aminolevulinate synthase	93.89	89.73
hemH		87.46	77.19
leuC	Leucine synthesis	96.58	95.3
leuB		96.76	89.19
phbB	Poly-beta-hydroxybutyrate (PHB) biosynthesis	96.68	87.5
phbC		82.37	71.45
dme	NAD-dependent malic enzyme	95.58	87.74
pckA	phosphoenolpyruvate carboxykinase	98.51	92.94
	**Transporter genes of cS23321**		
	**Description**	**cDOA9 (%)**	**cORS278 (%)**
mdtA		48.63	49.45
mdtB	Multidrug efflux	68.02	69.61
mdtC		94.2	85.78
dctA	Dicarboxylic acid transport	96.4	59.44
pstA		91.46	88.61
pstB	Pi transporter	94.86	87.57
pstC		94.36	90.5
groEL1,4,5	Chaperonin GroEL	94.25	90.91
groES1		93.27	86.54
groES5	Co-chaperonin GroES	93.88	90.82
groES4		92.31	83.65

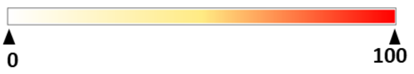

Supplement: Supplementary file 1 — Supplementary Material [file 37_22008_s1.pdf]
